# Synergistic Effect of Calcination Temperature and Silver Doping on Photocatalytic Performance of ZnO Material

**DOI:** 10.3390/ma18143362

**Published:** 2025-07-17

**Authors:** K. Kusdianto, Nurdiana Ratna Puri, Manabu Shimada, Suci Madhania, Sugeng Winardi

**Affiliations:** 1 Chemical Engineering Department, Institut Teknologi Sepuluh Nopember, Kampus ITS Sukolilo, Surabaya 60111, Indonesia; 7008232003@student.its.ac.id (N.R.P.); suci@its.ac.id (S.M.); swinardi@chem-eng.its.ac.id (S.W.); 2 Chemical Engineering Program, Graduate School of Advanced Science and Engineering, Hiroshima University, 4-1, Kagamiyama 1-Chome, Higashi-Hiroshima 739-8527, Hiroshima, Japan; smd@hiroshima-u.ac.jp

**Keywords:** composite, ultrasonic spray pyrolysis, photocatalytic, gas-phase

## Abstract

Ag-doped ZnO is a promising photocatalyst. However, the combined influence of the Ag doping concentration and furnace temperature has not been adequately explored, hindering the optimization of ZnO/Ag materials for practical applications. In this study, ZnO/Ag materials were synthesized via ultrasonic spray pyrolysis by systematically varying both the furnace calcination temperature and the Ag doping concentration. The synthesized materials were analyzed through a range of spectroscopic methods to investigate their structural, morphological, and surface characteristics. Their photocatalytic activity was assessed by monitoring the degradation of methylene blue (MB) under ultraviolet light exposure. The findings indicate that the ZnO sample that was calcined at 400 °C exhibited the highest degradation efficiency among the undoped samples, which can be attributed to its submicron particle size, moderate crystallinity, and high surface hydroxylation. The sample with 5-wt% Ag doping achieved enhanced performance, demonstrating the best photocatalytic activity (65% MB degradation). This improvement was attributed to the synergistic effects of surface plasmon resonance and optimized interaction between the Ag nanoparticles and surface hydroxyl groups. Excessive Ag loading (10 wt%) led to reduced activity owing to potential agglomeration and recombination centers. These results highlight the critical role of both the thermal and chemical parameters in tailoring ZnO-based photocatalysts for wastewater treatment applications.

## 1. Introduction

Zinc oxide (ZnO) is a wide-bandgap semiconductor (~3.37 eV) that has attracted growing interest owing to its excellent physicochemical stability, high electron mobility, non-toxicity, and strong photocatalytic potential [1,2,3,4]. These attributes make ZnO highly relevant for various applications, including photovoltaics [5], gas sensors [6], and, more importantly, the photocatalytic degradation [2,7] of organic pollutants in wastewater treatment. When exposed to ultraviolet (UV) light, ZnO produces electron–hole pairs that trigger the generation of reactive oxygen species (ROS), enabling the breakdown of harmful organic contaminants into less toxic products [8].

However, the practical efficiency of ZnO as a photocatalyst remains limited by its relatively low absorption in the visible light region and rapid recombination of photogenerated charge carriers [9]. To overcome these drawbacks, several methods have been applied, including doping [10], heterojunction engineering [11], and composite structures [12]. Among these methods, doping offers a more straightforward and controllable means of tailoring electronic structures and enhancing visible light absorption while maintaining the intrinsic crystal structure of ZnO. Unlike heterojunctions or composites—which often require complex multi-step fabrication and can introduce interfacial defects—doping enables uniform modification at the atomic level and is well-suited for cost-effective, scalable synthesis methods such as spray pyrolysis. Among the various metal dopants used to enhance ZnO, silver (Ag) offers distinct advantages, including its ability to induce localized surface plasmon resonance (LSPR) [13], enhance visible light absorption, and promote the effective separation of photogenerated charge carriers by acting as an electron sink. In a study conducted by Hussain et al. [10], the influence of the Ag doping concentration in the 1–6% range on the properties of ZnO was investigated; however, their work did not explore higher doping levels.

Besides doping, the method that is used to synthesize ZnO can also play a key role in determining its photocatalytic performance, as it can influence important material properties such as the particle size, shape, crystallinity, and surface area. These properties can directly affect how well the material works as a photocatalyst. Among the various synthesis methods available, spray pyrolysis stands out as a promising option, because it is simple, continuous, and requires just a single step [14]. Unlike conventional methods such as sol–gel [15], hydrothermal [16], or precipitation [17] methods—which typically involve multiple stages (e.g., aging, washing, drying, calcination)—spray pyrolysis enables better control over the composition and particle formation and is more amenable to scalable, industrial production [14].

Several previous studies have successfully fabricated Ag-doped ZnO (ZnO/Ag) [15,16,17,18,19,20]. However, most of these studies have focused primarily on general observations of the dopant-induced enhancements, without systematically examining how specific synthesis parameters—particularly the doping concentration and processing temperature—can affect the physicochemical properties and photocatalytic behavior of the material. Moreover, the majority of prior work employed liquid-phase synthesis methods—such as sol–gel or hydrothermal techniques—which generally require large amounts of solvent, involve multiple processing steps (e.g., aging, washing, drying, and calcination), and often result in products with lower purity and less uniformity. In particular, the combined influence of the Ag doping concentration and furnace temperature—two key parameters that govern nucleation, crystallinity, and defect formation during spray pyrolysis—has not been adequately explored. This lack of understanding hinders the ability to fully optimize ZnO/Ag materials for practical applications, particularly in wastewater treatment, where the material performance must be balanced with a cost-effective and scalable synthesis method.

This study investigated the characteristics of Ag-doped ZnO nanoparticles that were synthesized via ultrasonic spray pyrolysis (USP) by varying the furnace temperature (400, 500, 600, and 700 °C) and Ag doping concentration (1, 5, and 10 wt%). Characterization was performed to investigate the structural and morphological features of the prepared material, using X-ray diffraction (XRD), Fourier transform infrared spectroscopy (FTIR), and scanning electron microscopy (SEM). Moreover, the photocatalytic performance of the nanoparticles was evaluated by monitoring the degradation of methylene blue (MB) dye under UV light irradiation. While this study focused on optimizing photocatalytic efficiency through structural and process parameter tuning, it should be acknowledged that an analysis of potential intermediate products was not conducted.

## 2. Materials and Methods

### 2.1. Preparation Methods

**Figure 1:**
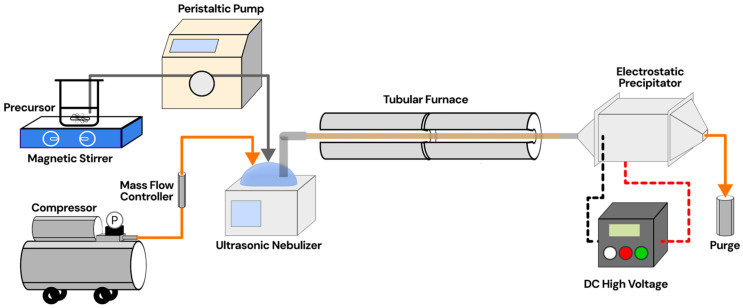
Experimental setup of particle synthesis using the ultrasonic spray pyrolysis method.

ZnO/Ag nanoparticles were synthesized using a USP system (laboratory made) based on procedures adapted from our previous study [21]. A schematic of the proposed system is presented in Figure 1. Here, the Omron NE-U17 was used as an atomizer. Zinc acetate dihydrate (Zn(CH_3_COO)_2_·2H_2_O, 99.5%, Merck, Darmstadt, Germany) was used as the zinc precursor and dissolved in distilled water to prepare a 0.1 M aqueous solution. To ensure complete dissolution and homogeneity, the solution was ultrasonicated for 30 min. The resulting precursor solution was then continuously pumped to the nebulizer using a peristaltic pump (EYELA MP-3N, Tokyo, Japan).

The precursor solution was atomized into fine droplets using an ultrasonic nebulizer, with these droplets being carried into a horizontal tubular furnace (custom length: 80 cm) using a carrier gas at a total flow rate of 2 L/min. The furnace was operated at varying temperatures (400, 500, 600, and 700 °C) to facilitate rapid solvent evaporation and thermal decomposition of the precursor, leading to the formation of ZnO-based nanoparticles. To avoid condensation and ensure effective particle collection, the aerosol stream was directed into an electrostatic precipitator, maintained at 120 °C and operating at a voltage of 40 kV. Silver doping was achieved by adding silver nitrate (AgNO_3_, 99.5%, Merck, Germany) to the precursor solution, resulting in final Ag concentrations of 1, 5, and 10 wt%.

### 2.2. Material Characterization

The morphological features of the synthesized ZnO and ZnO/Ag nanoparticles were examined using SEM (FlexSEM 1000, Hitachi High Technologies, Schaumburg, IL, USA) to observe the surface structure, particle shape, and degree of agglomeration. To gain a clearer insight into the surface morphology, FE-SEM (Hitachi Regulus 8220, Tokyo, Japan) analysis was subsequently carried out. The crystalline structure and phase composition were analyzed using the X’Pert Pro multi-purpose XRD system (PANalytical, Shanghai, China), operated with Cu Kα radiation (*λ* = 1.5406 Å). The diffraction patterns were recorded in the 2θ range of 20–80°, and the crystallite sizes were estimated using the Scherrer equation:

(1) $$D=\frac{k\lambda }{B\cos\theta }$$

where *k* denotes a constant (*k* = 0.9), λ denotes the X-ray wavelength (*λ* = 1.5406 Å), *B* denotes the full width at half maximum (FWHM) of the peak, and θ denotes the XRD peak angle [22].

To identify the presence of functional groups and to investigate the chemical bonding within the samples, FTIR (Thermo Scientific Nicolet iS10, Waltham, MA, USA) spectroscopy was performed in the 4000–400 cm^−1^ spectral range using the KBr pellet method.

### 2.3. Photocatalytic Test

The photocatalytic performance of the synthesized nanoparticles was evaluated using MB as a model organic dye pollutant. A 40 mL aqueous solution of MB was prepared with a concentration of 10 ppm, and 60 mg of the photocatalyst material was dispersed into the solution. Prior to irradiation, the suspension was stirred in the dark for 30 min, as preliminary tests indicated that the adsorption–desorption equilibrium between the dye molecules and catalyst surface was effectively achieved within this period. A 10 ppm MB concentration was selected to ensure measurement accuracy within the spectrophotometer’s linear range while maintaining consistency with standard conditions used in previous photocatalytic studies

The photocatalytic degradation process was then conducted under UV light irradiation for 75 min. At 15 min intervals, aliquots were withdrawn and centrifuged at 5000 rpm to separate the catalyst particles from the solution. The transparent supernatant was analyzed using a UV–Vis spectrophotometer (Thermoscientific Genesys, Waltham, MA, USA) at the peak absorption wavelength of MB (*λ_max_* = 664 nm). Changes in the absorbance values were used to monitor the concentration of the remaining dye over time.

## 3. Results and Discussion

### 3.1. Effect of Calcination Temperature on ZnO’s Properties and Photocatalytic Activity

To investigate the effect of the furnace temperature on the structural and morphological properties of the synthesized materials, the ZnO materials were fabricated at various calcination temperatures—400, 500, 600, and 700 °C. The furnace temperature is a crucial parameter in the spray pyrolysis process, as it can directly affect the solvent evaporation rate, precursor decomposition, and subsequent nucleation and growth of the particles. A temperature that is too low can result in incomplete precursor decomposition and poor crystallinity, whereas excessively high temperatures can lead to particle sintering and undesirable agglomeration [23].

**Figure 2:**
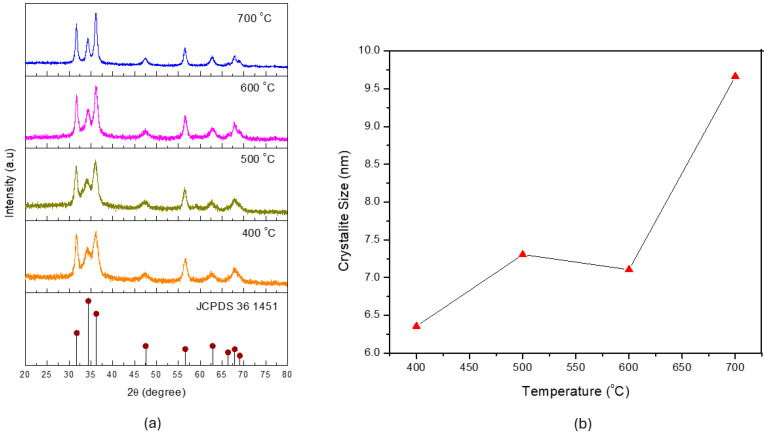
(**a**) XRD spectra and (**b**) cumulative crystallite size of the ZnO material when fabricated at various furnace temperatures.

Figure 2a shows the XRD patterns of the ZnO material when synthesized at various furnace temperatures (400–700 °C). All diffraction peaks were consistent with the hexagonal wurtzite ZnO phase (JCPDS card No. 36-1451), and no secondary phases could be detected, indicating high phase purity across all samples. As the temperature increased, the diffraction peaks became sharper and more intense, particularly at 700 °C. This trend suggests improved crystallinity and grain growth owing to the enhanced thermal energy facilitating more complete crystal formation and particle coalescence.

To quantitatively assess the crystallinity, the crystallite size was calculated using the Debye–Scherrer equation (Equation (1)), the results of which are shown in Figure 2b. Interestingly, although the crystallite size increased from 400 to 500 °C, a slight decrease was evident at 600 °C, before a substantial jump occurred at 700 °C. This indicates that at sufficiently high temperatures, the thermal energy overcomes prior barriers to crystal growth. The reduction at 600 °C could be attributed to a recrystallization or restructuring process. At 600 °C, the thermal energy could have induced partial dissolution or rearrangement of previously formed crystallites, resulting in the fragmentation of larger crystals into smaller domains. Such restructuring could temporarily reduce the average crystallite size before subsequent growth resumed at higher temperatures [24,25]. The increase in crystallite size at 700 °C could be attributed to the enhanced atomic mobility at higher temperatures, facilitating the coalescence and growth of existing crystallites. This stage is typically dominated by grain growth rather than nucleation, allowing for the formation of larger and more ordered crystal domains [25].

**Figure 3:**
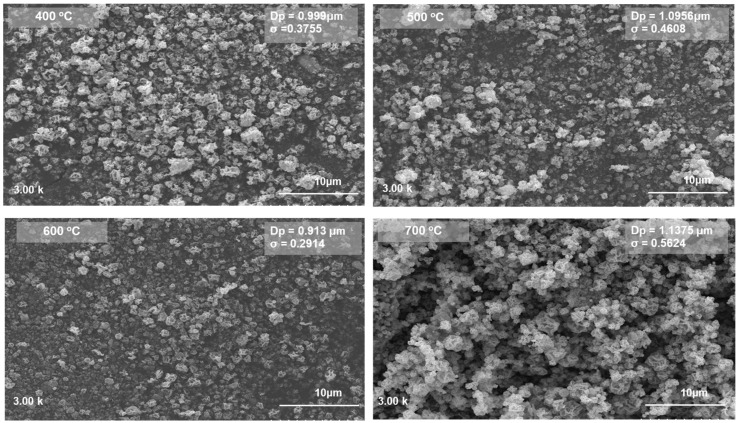
SEM images of the ZnO material, fabricated at various temperatures.

The surface morphology of the ZnO particles that were synthesized at different temperatures was further investigated using SEM analysis, as shown in Figure 3. The particle size distribution and agglomeration behavior varied considerably with increasing synthesis temperatures. At 400 °C, the particles were relatively uniform and well-dispersed, with an average size of 0.999 µm and moderate size distribution (*σ* = 0.3755). Increasing the temperature to 500 °C slightly enlarged the particles (1.0956 µm) and broadened the distribution (*σ* = 0.4608), indicating early-stage agglomeration and coalescence. Moreover, at 600 °C, the average particle size unexpectedly decreased to 0.913 µm, with a narrower distribution (*σ* = 0.2914), suggesting a thermally driven imbalance between the nucleation and growth mechanisms. At this temperature, rapid decomposition of the precursor could lead to a high nucleation rate, generating many smaller crystallites. Moreover, the limited time or insufficient energy for substantial crystal growth could induce lattice disorder, contributing to the evident reduction in crystallite size and increased micro-strain [22,23,26]. At 700 °C, the particles grew larger (1.1375 µm) and more densely packed, with the broadest size distribution (*σ* = 0.5624), owing to enhanced atomic diffusion, which facilitated the migration of atoms to existing crystal surfaces and promoted grain coarsening—consistent with the highest crystallinity seen in the XRD results [27]. It is worth noting that at temperatures up to 600 °C, the ZnO particles remained predominantly in the submicron range (<1 µm), which is particularly desirable for applications requiring a high surface area, such as photocatalysis or sensor development.

It is also worth addressing the apparent discrepancy between Figure 2 and Figure 3 regarding the particle size of the synthesized ZnO materials. As shown in Figure 2, the crystallite size calculated from the XRD analysis using the Scherrer equation was in the nanometer range (6.25–9.5 nm), representing the size of the coherent crystalline domains within the ZnO structure. By contrast, Figure 3 shows SEM images exhibiting particle sizes in the micrometer range, which reflect the morphological aggregates that were formed during the spray pyrolysis and subsequent calcination processes. This difference in scale is to be expected, as primary nanocrystallites tend to agglomerate to reduce the surface energy, leading to the formation of larger secondary structures while retaining their nanocrystalline domains within. A similar observation was reported by Agarwal et al. (2019) [28], where ZnO materials exhibited nanometer-scale crystallite sizes alongside micrometer-scale morphological aggregates in SEM images.

**Figure 4:**
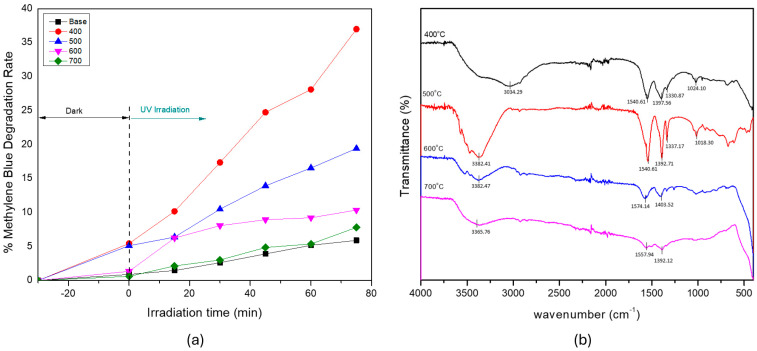
(**a**) Photocatalytic and (**b**) FTIR results for the ZnO material, fabricated at various temperatures.

Figure 4a presents the photocatalytic degradation of MB under UV irradiation using Ag-doped ZnO nanoparticles synthesized at different calcination temperatures (400, 500, 600, and 700 °C), along with the undoped ZnO sample (Base). Among the tested samples, the material that was calcined at 400 °C exhibited the highest photocatalytic activity, followed by those that were calcined at 500 °C, 600 °C, and 700 °C, respectively. This trend highlights the critical influence of the calcination temperature on the structural and surface properties of the photocatalyst.

The enhanced performance of the 400 °C sample could be attributed to its relatively small crystallite size (6.25 nm), which contributed to a higher specific surface area and more active sites for photocatalytic reactions. Additionally, this temperature likely maintained a high concentration of surface hydroxyl groups, as indicated by the broad and intense O–H stretching band (3200–3600 cm^−1^) that is evident in the FTIR spectrum (Figure 4b). These surface hydroxyls facilitate the formation of hydroxyl radicals (•OH) under UV irradiation, which are essential for the degradation of organic pollutants [29]. Notably, the intensity of this O–H band was highest in the sample that was calcined at 400 °C, indicating the greater availability of surface hydroxyl groups, which was consistent with its superior photocatalytic performance. The peak in the 1600–1400 cm^−1^ region is typically associated with O–H bending and C = O stretching vibrations, which can originate from residual organic species. In the fingerprint region (400–650 cm^−1^), the absorption band around 420 cm^−1^ can be assigned to Zn–O stretching vibrations, confirming the presence of the ZnO phase [10]. This band is clearly visible in all samples, although its intensity can vary slightly owing to differences in the crystallinity and particle aggregation that are induced by the calcination temperature.

As the calcination temperature increased to 500 and 600 °C, the crystallite size increased to 7.25 nm and slightly decreased to 7.1 nm, respectively. Although these sizes remained within the nanoscale range, the gradual loss of surface hydroxyl groups—as evidenced by the weakening O–H bands in the FTIR spectra—could reduce the photocatalytic efficiency. At 700 °C, the photocatalytic activity dropped considerably, corresponding to the largest crystallite size (9.5 nm) and weakest O–H signal. The larger particle size likely resulted in a lower surface area, reduced surface reactivity, and diminished availability of hydroxyl groups, all of which negatively impact the photocatalytic performance.

These results indicate that an optimal balance between crystallinity, surface hydroxyl density, and particle size is essential for maximizing the photocatalytic activity. The sample that was calcined at 400 °C demonstrated this balance most effectively, making it the most suitable condition for synthesizing high-performance Ag-doped ZnO photocatalysts via spray pyrolysis. Additionally, the inclusion of a photolysis control using the undoped ZnO base sample without UV illumination confirmed that the observed MB degradation was not solely owing to adsorption or spontaneous breakdown. The major difference between the irradiated and non-irradiated samples further verified the role of photocatalytic activity driven by UV light exposure.

**Figure 5:**
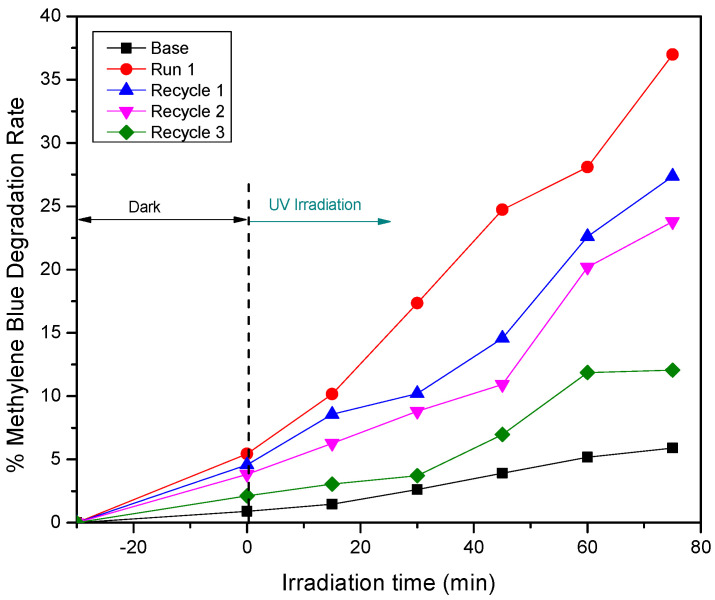
Reusability performance of the ZnO photocatalyst (sample calcined at 400 °C) for methylene blue (MB) degradation under UV irradiation over three consecutive cycles.

Given that the photocatalyst that was calcined at 400 °C exhibited the highest photocatalytic performance among all the tested temperatures, a reusability test was conducted on this optimal sample to evaluate its operational stability and potential for repeated use in practical wastewater treatment applications. As shown in Figure 5, the Ag-doped ZnO photocatalyst was subjected to three consecutive MB photocatalytic degradation cycles under UV irradiation. The results demonstrated that although the degradation efficiency gradually decreased over successive cycles, the photocatalyst maintained considerable activity, achieving approximately 12% degradation after the third cycle.

This decline in performance could be attributed to partial photocorrosion, particle agglomeration reducing the effective surface area, or accumulation of the degradation by-products on active sites, which could inhibit further photocatalytic reactions. Despite these factors, the material’s ability to sustain photocatalytic activity over multiple cycles indicated its promising reusability and stability, underscoring its feasibility for scalable wastewater treatment applications.

**Figure 6:**
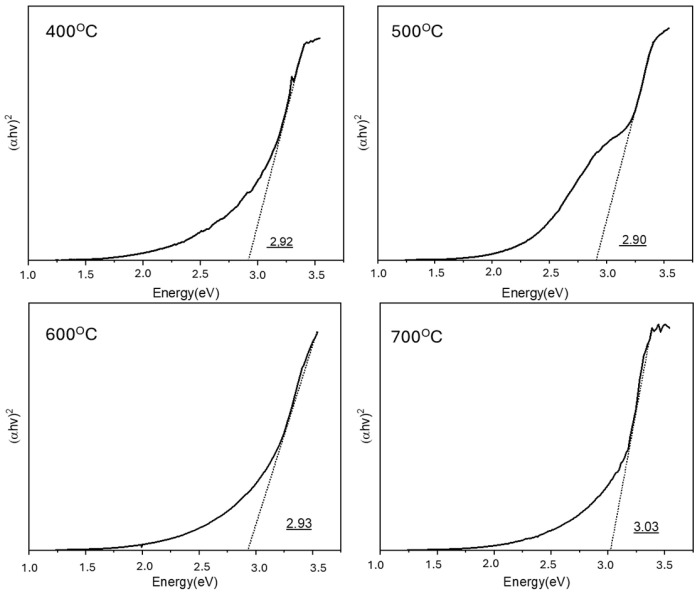
Band gap analysis of the ZnO photocatalyst at various temperatures.

Figure 6 shows Tauc plots for the ZnO samples that were calcined at 400, 500, 600, and 700 °C, revealing band gap energies of 2.924, 2.885, 2.847, and 3.000 eV, respectively. These values were slightly lower than the typical band gap of pristine ZnO, which is generally reported to be approximately 3.2–3.37 eV. The initial decrease in the band gap with increasing calcination temperatures (400–600 °C) can be attributed to grain growth and a reduction in defect-related localized states, leading to band gap narrowing via improved crystallinity and reduced quantum confinement effects.

The subsequent increase in the band gap at 700 °C could be associated with several factors. First, excessive thermal treatment could induce lattice strain relaxation and alter the electronic band structure, causing a slight blue-shift in the band gap. Second, higher calcination temperatures could reduce the oxygen vacancy concentrations and other defect states that typically contribute to band gap narrowing, thus shifting the band gap toward the intrinsic value of ZnO. Additionally, the evident increase in crystallite size at 700 °C could reduce the density of localized defect states near the conduction band edge, further contributing to the evident band gap widening.

These observations underscore the critical role of calcination temperature in tuning the optical properties of ZnO. As the band gap energy directly influences the generation of electron–hole pairs and light absorption capabilities, controlling the calcination temperature provides a strategy to optimize the photocatalytic performance of ZnO-based materials under UV irradiation for wastewater treatment applications.

### 3.2. Effect of Silver Dopant Concentration on ZnO’s Properties and Photocatalytic Activity

**Figure 7:**
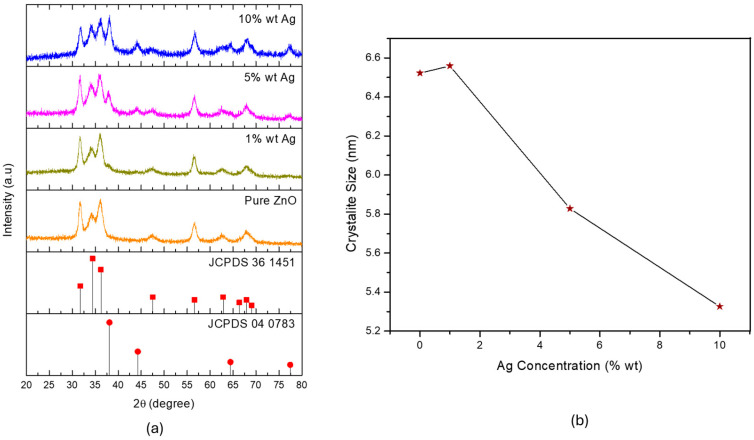
(**a**) XRD spectra and (**b**) cumulative crystallite size of the ZnO material when fabricated at various silver dopant concentrations.

Following the identification of 400 °C as the optimal calcination temperature for ZnO synthesis, further investigation was conducted by introducing silver (Ag) as a dopant to tailor the material’s properties. Ag was incorporated into ZnO with varying molar ratios of 1%, 5%, and 10%, the resulting crystal structures of which were analyzed using XRD, as shown in Figure 7a.

All doped samples exhibited diffraction peaks that were characteristic of the hexagonal wurtzite ZnO phase (JCPDS No. 36-1451), indicating that Ag doping did not alter the primary crystalline structure of ZnO (Figure 7). The dominant peaks evident at 2θ ≈ 31.8°, 34.4°, and 36.3°—corresponding to the (100), (002), and (101) planes, respectively—remained consistent across all doping levels. According to the JCPDS, all synthesized samples exhibited a characteristic hexagonal wurtzite crystal structure, representing the most thermodynamically stable form of ZnO. Additionally, the XRD analysis revealed that the resulting nanocomposites exhibited a high degree of crystallinity. The most prominent diffraction peak appeared at 36.2°, with additional peaks at 38.1°, 44.3°, 64.4°, and 77.5° emerging when the Ag content reached or exceeded 5 wt%. These peaks could be attributed to the (111), (200), (220), and (300) planes of metallic silver, indicating the presence of Ag phases within the ZnO–Ag nanocomposite system [30].

Moreover, the crystallite size could be calculated using the Debye–Scherrer calculations, as plotted in Figure 7b. The crystallite size of the ZnO increased with 1 wt% Ag doping, suggesting that a small amount of Ag^+^ could have been effectively incorporated into the ZnO lattice, promoting grain growth. However, further increases in Ag content to 5 and 10 wt% led to a gradual reduction in the crystallite size. This trend could be attributed to the excessive incorporation of larger Ag^+^ ions, which caused considerable structural defects, thereby inhibiting further crystallite growth [9,29,30].

**Figure 8:**
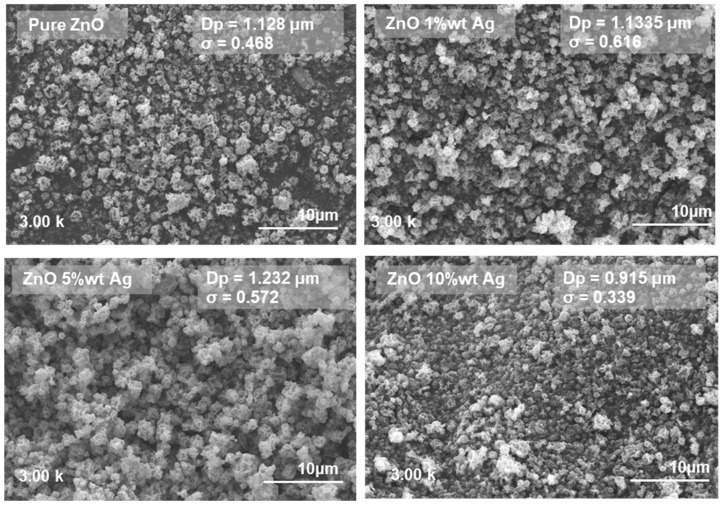
SEM images of the ZnO material, fabricated with various silver dopant concentrations.

The surface morphologies of the pure and Ag-doped ZnO samples were analyzed using SEM, the results of which are presented in Figure 8. The undoped ZnO sample exhibited a relatively uniform spherical morphology with noticeable agglomeration and an average particle diameter (*D_p_*) of 1.128 µm, with a distribution width (σ) of 0.468. Upon doping with 1 and 5 wt% Ag, the particle sizes increased to 1.147 (*σ* = 0.520) and 1.232 µm (*σ* = 0.572), respectively. However, further increasing the Ag doping to 10 wt% resulted in a marked decrease in particle size to 0.915 µm (*σ* = 0.339).

**Figure 9:**
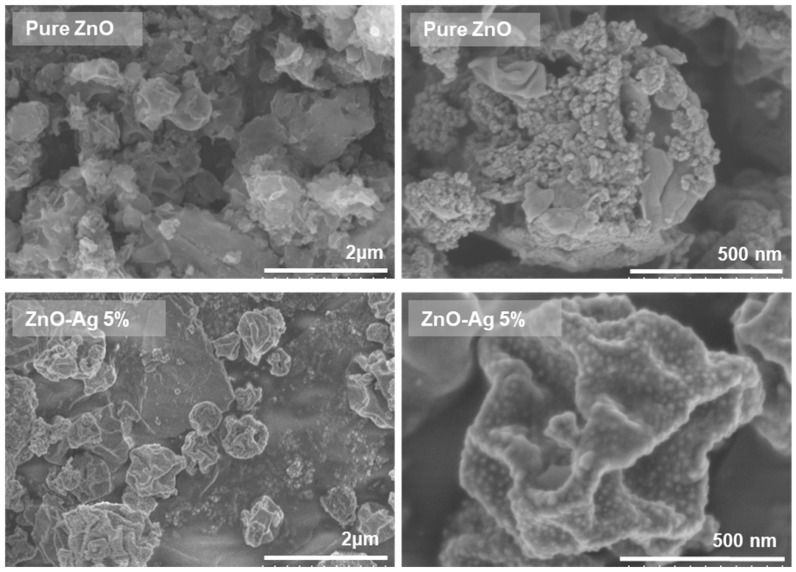
FE-SEM images of pure ZnO and ZnO-Ag 5% wt.

The initial increase in particle size from the pure ZnO to 5-wt% Ag could be attributed to the role of Ag as a heterogeneous nucleation promoter, facilitating crystal growth during the calcination process. The Ag atoms could substitute into the Zn^2+^ sites, thereby altering the growth kinetics and promoting coarsening of the ZnO particles. This phenomenon has been reported in similar systems, where moderate dopant levels induced grain boundary migration and growth [31,32,33]. However, at a higher Ag concentration (10 wt%), the particle size decreased considerably, likely because of dopant saturation, whereby excessive Ag inhibited grain growth by acting as a barrier at the grain boundaries [8,9,31]. These effects are likely associated with the suppression of particle agglomeration that occurs at elevated dopant concentrations. It should be noted that SEM analysis in this study was primarily employed to capture the overall morphology and to provide an estimation of the particle sizes at the microscale, while more detailed nanostructural analysis was performed using FE-SEM, as presented in Figure 9. The nanostructural morphology of the synthesized samples was further examined using FE-SEM, as displayed in Figure 9, focusing on undoped ZnO and 5 wt% Ag-doped ZnO as representative samples. The pure ZnO sample exhibits a more aggregated and compact morphology with dense clusters, indicating strong particle–particle adhesion during the synthesis process. In contrast, the ZnO-Ag 5% sample shows a relatively less agglomerated structure with clearer particle boundaries, suggesting that Ag incorporation helps to reduce excessive aggregation. Additionally, the presence of fine bright spots that are uniformly distributed on the surface of ZnO-Ag 5% can be observed, which are attributed to Ag nanoparticles successfully attaching to the ZnO surface. These features confirm that Ag particles are well-dispersed and anchored on the ZnO, potentially functioning as electron sinks to enhance charge separation during photocatalysis. This observation is consistent with previous reports, such as the study by Zhu et al. [34], who also found that Ag being deposited on ZnO surfaces resulted in distinct dot-like structures on the surface and reduced agglomeration, correlating with improved photocatalytic properties. These findings provide morphological evidence supporting the functional role of Ag in modifying surface properties and facilitating photocatalytic enhancement in ZnO-based systems.

**Figure 10:**
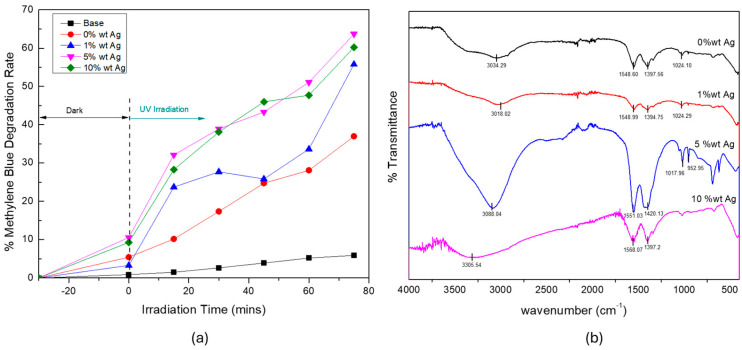
(**a**) Photocatalytic and (**b**) FTIR results for the ZnO material when fabricated at various silver dopant concentrations.

To evaluate the photocatalytic efficiency of the synthesized materials, the decomposition of the MB dye was monitored under UV irradiation. As shown in Figure 10a, the degradation efficiency varied considerably with the Ag doping concentration. The 5-wt% Ag-doped ZnO sample exhibited the highest photocatalytic activity, achieving a degradation efficiency of 65%, followed by the 10-wt%, 1-wt%, and undoped ZnO (0 wt%) samples. This enhancement in photocatalytic activity at 5-wt% Ag could be attributed to several synergistic effects. By integrating Ag into the ZnO matrix, the charge separation efficiency was enhanced, as the Ag functioned as an electron reservoir, effectively lowering the recombination rate of the photogenerated electron–hole pairs [35]. Notably, this performance is comparable or superior to previous studies, such as those by Mirzaeifard et al. [36], who reported 53% degradation, and Uribe-López et al. [37], who reported 48% degradation on mineralized pollutant water under 120 min of irradiation, confirming the competitive efficiency of the synthesized Ag-doped ZnO under shorter irradiation times in this work. The optimal Ag concentration (5 wt%) likely provided an ideal balance between the improved charge transport and light absorption without introducing excessive recombination centers or surface coverage. The slight decrease in activity at a higher Ag loading (10 wt%) could be because of the excessive presence of Ag species on the ZnO surface, which could block active sites or act as recombination centers when surpassing a critical threshold [38].

At lower concentrations (1 wt%), the amount of Ag could be insufficient to notably improve the charge separation or extend the light absorption. Moreover, the pure ZnO (0 wt%) exhibited the lowest degradation efficiency owing to the rapid recombination of the photo-induced carriers [38]. Interestingly, the superior performance of the 5-wt% Ag-doped ZnO sample was further supported by the FTIR analysis (Figure 10b), which revealed a noticeably broader absorption band of ~1600 cm^−1^, which corresponded to the bending vibration (δ–OH) of surface hydroxyl groups and/or adsorbed water molecules [17]. The evident notable broadening in this sample suggests the presence of a heterogeneous chemical environment, possibly because of the increased interaction between the Ag nanoparticles and surface –OH groups [39].

Surface hydroxyl groups are considered essential in photocatalytic processes, serving as initial sites for the formation of ROS—such as hydroxyl radicals (•OH)—which are instrumental in the dye degradation. The broader δ–OH band observed in the 5-wt% Ag sample implied an increase in surface hydroxyls, which could facilitate more effective ROS formation during UV irradiation [39].

**Figure 11:**
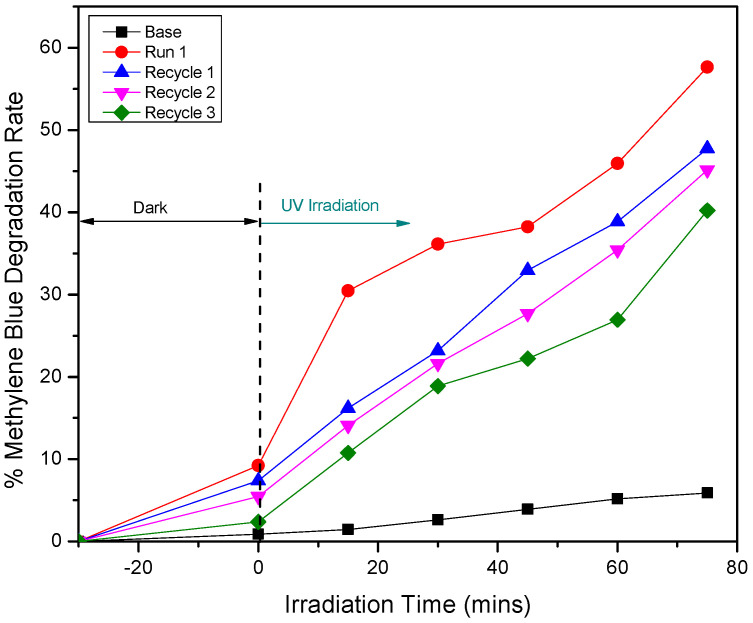
Reusability performance of the Ag-doped ZnO photocatalyst sample that was calcined at 400 °C for methylene blue (MB) degradation under UV irradiation over three consecutive cycles.

Given the promising photocatalytic activity that was evident for the 5% Ag-doped ZnO fabricated at 400 °C, under UV irradiation, reusability tests were performed to evaluate its operational stability across multiple cycles. As shown in Figure 11, the photocatalyst was subjected to three consecutive MB degradation cycles under identical UV irradiation conditions. The initial run (Run 1) exhibited the highest photocatalytic degradation efficiency, achieving approximately 57% after 75 min of irradiation. In subsequent cycles (Recycle 1, Recycle 2, and Recycle 3), a gradual decrease in photocatalytic efficiency was evident, yet the material consistently maintained effective degradation capabilities across all cycles. By the third cycle, the photocatalyst retained a degradation efficiency of approximately 38%, indicating that the material remained active and operational for extended use. Notably, the degradation efficiency in the third cycle remained higher than that of the undoped ZnO (base) material under the same conditions, indicating the beneficial role of Ag doping in sustaining the photocatalytic performance, even after repeated use.

**Figure 12:**
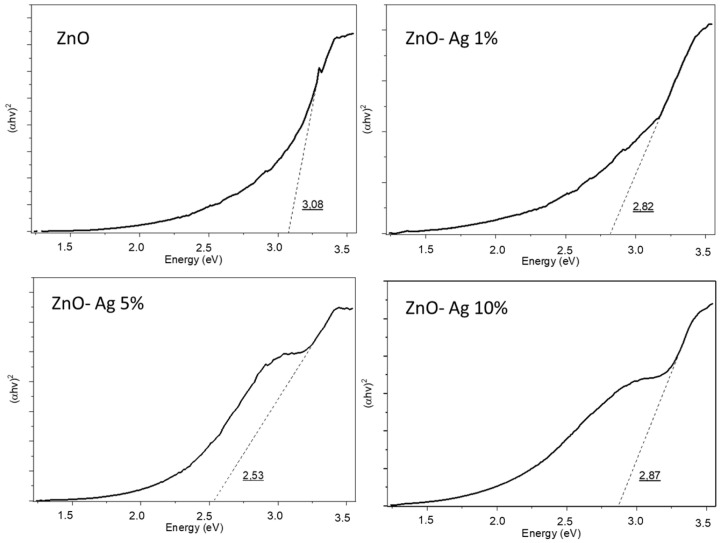
Band gap analysis of the ZnO–Ag materials at different doping concentrations.

The band gap energies of the pure ZnO and Ag-doped ZnO with different doping concentrations are shown in Figure 12. The band gap of the pristine ZnO was measured to be 3.08 eV, which was consistent with the reported band gap range for ZnO (3.0–3.2 eV). Upon doping with Ag, the band gap values decreased to 2.82 eV for the 1-wt% Ag, further reduced to 2.53 eV for the 5-wt% Ag, and slightly increased to 2.87 eV for the 10-wt% Ag. The trend of band gap narrowing with increasing Ag contents up to 5 wt% could be attributed to the introduction of impurity levels and defect states within the band structure of the ZnO, which facilitated band tailing and effectively reduced the optical band gap [2,3]. The incorporation of Ag ions created localized energy states near the conduction band, enhancing the absorption of lower-energy photons and improving the visible-light-harvesting capability, which is advantageous for photocatalytic applications [4].

Interestingly, at a higher doping concentration of 10-wt% Ag, the band gap increased to 2.87 eV. This increase can result from the excessive Ag loading leading to the formation of Ag clusters on the ZnO surface, which can act as recombination centers rather than contributing to the narrowing of the band gap [5]. Furthermore, at higher doping concentrations, the interaction between the Ag and ZnO can reach a saturation limit, with the effective Ag contribution to band tailing becoming limited, thus slightly increasing the band gap [6]. Overall, the reduction in the band gap with Ag doping up to an optimal concentration suggests enhanced photon absorption in the visible region, which can improve photocatalytic activity. However, excessive doping can induce adverse effects owing to recombination center formation and surface agglomeration, emphasizing the importance of optimizing the dopant concentration to balance the improved light absorption with charge carrier dynamics in Ag-doped ZnO photocatalysts.

**Figure 13:**
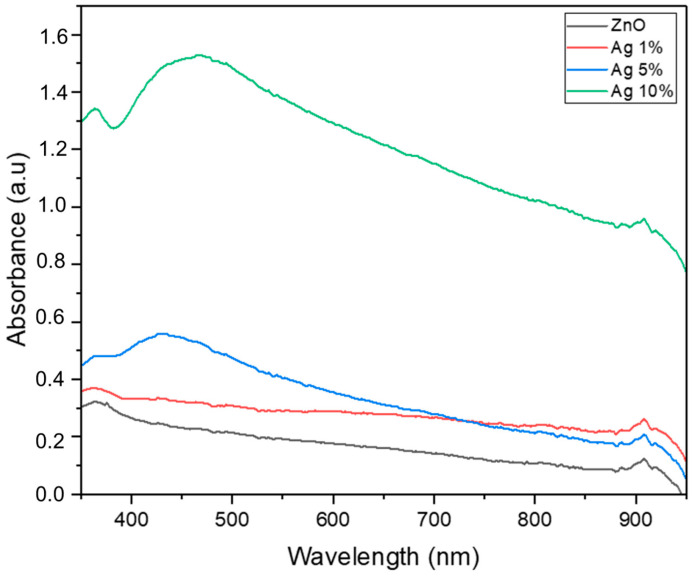
Absorbance spectra of the ZnO–Ag nanoparticles with different doping concentrations.

Figure 13 shows the UV–Vis absorption spectra of the pristine ZnO and Ag-doped ZnO with 1%, 5%, and 10% Ag doping concentrations. The pure ZnO exhibited strong absorption in the UV region (~370 nm) with a rapid decrease in the visible region, indicating its wide-band-gap nature, which restricts its light-harvesting capability under visible light. Upon Ag doping, the absorption spectra displayed a clear enhancement in the visible region (400–600 nm), particularly evident for the 5% and 10% Ag-doped samples. This enhanced visible light absorption could be attributed primarily to the surface plasmon resonance (SPR) effect of Ag nanoparticles, which can induce strong localized electromagnetic fields upon light excitation, thereby increasing the light-harvesting ability of the ZnO matrix in the visible region [1,2]. The presence of SPR is typically manifested as a broad absorption band in the visible region, as observed in the Ag 10% sample, which exhibited the highest absorbance enhancement.

Moreover, the increased visible light absorption with higher Ag contents suggests improved utilization of the solar spectrum, which is beneficial for photocatalytic applications. However, the variation in absorbance intensity among the different doping levels indicates that the dispersion and particle size of Ag within the ZnO matrix, along with possible agglomeration at higher doping levels, could influence the plasmonic behavior and thus the optical properties [3]. The SPR of Ag not only extends the light absorption into the visible range but also facilitates efficient charge separation by acting as an electron sink, reducing the recombination rate of photogenerated electron–hole pairs in the ZnO. This synergistic effect is expected to enhance the photocatalytic performance of Ag-doped ZnO under visible light irradiation [4]. In summary, the UV–Vis absorption spectra confirmed that Ag doping effectively modified the optical properties of the ZnO through SPR, enhancing its visible light absorption and potentially improving its photocatalytic activity under solar irradiation.

## 4. Conclusions

This study demonstrated the considerable influence of both the calcination temperature and silver doping concentration on the photocatalytic performance of ZnO nanoparticles that are synthesized via USP. The optimal calcination temperature was found to be 400 °C, yielding ZnO particles with a favorable submicron morphology and high surface hydroxyl content that contributed to the effective photocatalytic degradation of MB. Further enhancement could be achieved by doping with Ag, where the 5-wt% Ag-doped ZnO sample exhibited the highest degradation efficiency of 65%. This superior performance could be attributed to synergistic effects—including enhanced electron–hole separation owing to the Ag acting as an electron sink, LSPR effects, and increased surface reactivity (as confirmed by FTIR analysis). Specifically, the broadening of the δ–OH vibration band at ~1600 cm^−1^ suggested stronger interaction between the Ag and surface hydroxyl groups, which played a vital role in generating ROS during the photocatalysis process. Overall, the combination of optimized calcination conditions and appropriate Ag doping levels enabled the fabrication of ZnO-based photocatalysts with improved efficiency, offering enormous potential for environmental applications such as wastewater treatment.

## Data Availability

The original contributions presented in this study are included in the article. Further inquiries can be directed to the corresponding author.

## Abbreviations

The following abbreviations are used in this manuscript:

FTIR	Fourier transform infrared
FWHM	Full width at half maximum
LSPR	Localized surface plasmon resonance
MB	Methylene blue
ROS	Reactive oxygen species
SEM	Scanning electron microscopy
USP	Ultrasonic spray pyrolysis
UV	Ultraviolet
XRD	X-ray diffraction
